# The Role of Maternal Dietary Proteins in Development of Metabolic Syndrome in Offspring

**DOI:** 10.3390/nu7115460

**Published:** 2015-11-06

**Authors:** Alireza Jahan-Mihan, Judith Rodriguez, Catherine Christie, Marjan Sadeghi, Tara Zerbe

**Affiliations:** Department of Nutrition and Dietetics, Brook College of Health, University of North Florida, UNF Dr. Bldg 39, Room 3057A, Jacksonville, FL 32224, USA; jrodrigu@unf.edu (J.R.); c.christie@unf.edu (C.C.); n00961956@ospreys.unf.edu (M.S.); tjz1973@comcast.net (T.Z.)

**Keywords:** protein, fetal programming, metabolic syndrome

## Abstract

The prevalence of metabolic syndrome and obesity has been increasing. Pre-natal environment has been suggested as a factor influencing the risk of metabolic syndrome in adulthood. Both observational and experimental studies showed that maternal diet is a major modifier of the development of regulatory systems in the offspring *in utero* and post-natally. Both protein content and source in maternal diet influence pre- and early post-natal development. High and low protein dams’ diets have detrimental effect on body weight, blood pressure191 and metabolic and intake regulatory systems in the offspring. Moreover, the role of the source of protein in a nutritionally adequate maternal diet in programming of food intake regulatory system, body weight, glucose metabolism and blood pressure in offspring is studied. However, underlying mechanisms are still elusive. The purpose of this review is to examine the current literature related to the role of proteins in maternal diets in development of characteristics of the metabolic syndrome in offspring.

## 1. Introduction

Metabolic syndrome is a cluster of metabolic disorders and it is defined as a combination of central obesity and insulin resistance plus any two of the following four factors: Raised triglycerides, reduced HDL, raised blood pressure and raised fasting plasma glucose [1]. The prevalence of metabolic syndrome and obesity has been increasing since the mid-20th century [2]. It also affects between 22.9% of the US population and up to 36% of Europeans aged 40–55 [3,4]. However, the pathophysiology of metabolic syndrome is not fully understood. The overarching role of impaired insulin resistance and central obesity has been suggested [5]. More recently, an association between the pre-natal environment and the risk of metabolic syndrome in adulthood has been reported [6,7].

There is substantial evidence supporting the role of fetal and early post-natal nutrition in development of somatic structures, endocrine systems and homeostatic mechanisms of the fetus and infant. These effects can also influence the risk of obesity, hypertension, diabetes and other components of metabolic syndrome in later life [8,9,10]. The role of early ontogeny to later life health has been named fetal programming, which is defined as the process whereby a stimulus during a critical period of early development results in long-term physiological consequences [11,12].

Dietary proteins elicit a wide range of nutritional and biological functions. Beyond their nutritional role as the source of amino acids for protein synthesis, they are instrumental in the regulation of food intake, glucose and lipid metabolism, blood pressure, bone metabolism and immune function [13]. Physico-chemical properties, amino acid composition and bioactive peptides encrypted in amino acid sequences of proteins contribute to physiological functions of proteins [13]. Moreover, the role of proteins in development during pregnancy and early life has also been studied.

The role of low and high protein maternal diets in health outcome of the offspring has been examined extensively particularly in animals [12,14,15,16,17]. Both low and high protein diets during pregnancy influence body weight, blood pressure and metabolic and intake regulatory systems in the offspring. Low protein maternal diets increase blood pressure [15,17], body weight [14] and adiposity [18] whilst high protein maternal diets increase body weight [19], blood pressure and food efficiency [20] and decrease energy expenditure [21,22] in offspring of rats. Furthermore, the source of protein in a nutritionally adequate environment influences development of intake regulatory system and characteristics of metabolic syndrome in a sex-dependent manner in offspring of Wistar rats [23,24,25]. Food intake, body weight, fat pad mass, systolic and diastolic blood pressure, plasma homocysteine and hypothalamic gene expression of Agouti Related Protein (AgRP) were higher in offspring born to dams fed the soy protein-based diet while fasting blood glucose and Homeostatic Model of Assessment of Insulin Resistance (HOMA-IR) index were higher only in male offspring born to dams fed the soy protein-based diet compared with those born to dams fed the casein-based diet [23,24,25]. These results suggest that the effect of proteins in maternal diet on phenotype of offspring is beyond the effect of protein content of the diet. The underlying mechanisms are unclear at present but characteristics of proteins including their digestive kinetics, amino acid sequence and bioactive peptides encrypted in their amino acid sequence are potential factors determining the physiological functions of proteins.

Therefore, the purpose of this review is to discuss the role of proteins in maternal diets on development of characteristics of the metabolic syndrome in offspring.

The vast majority of experimental studies in this field have been conducted in animals (particularly rodents) and it can be due ethical reasons and also the hierarchical model of these studies investigating multiple generations. The rat constitutes a good experimental model for the studies of trophoblast invasion, which is intimately associated with the remodeling of maternal tissues during the normally progressing pregnancy but in comparison to human rodents have a shorter gestation period (21–23 days) [26]. It is critically important to be taken into account that rodents are born underdeveloped and many neurogenic events occur postnatally [27]. Humans, although considered an altricial species, are neutrally advanced at birth relative to the other species studied [27]. It is suggested that the gestational period of rats, corresponds to the first two trimesters of human gestation [28] and lactation in rodents corresponds to the 3rd trimester of pregnancy in humans, not to the humans’ breastfeeding period. Therefore, rodents are a good experimental model for studying pregnancy in human. Consequently, this review has more focus on animal studies.

## 2. Fetal Programming

The notion that the intrauterine environment may influence the development of the fetus is not novel. However, the concept that fetal development impacts on adult diseases has arisen relatively recently and led to a revival of interest in the influence of the *in utero* environment on the fetus and neonate [29]. Developmental plasticity allows the fetus to adapt tissue structure in response to environmental changes. The long-term post-natal consequences of developmental plasticity have been described by a number of terms including programming [30] and metabolic imprinting [31]. Programming is described as any situation where a stimulus or an insult during development induces a permanent physiological response [30] whilst, metabolic imprinting covers adaptive responses to specific nutritional conditions in early life that are characterized by a susceptibility limited to a critical developmental window in early life, a persistent effect lasting through adulthood, and a specific and measurable outcome [32].

In 1934, Kermack *et al.* reported a fall in death rates from all causes between 1751 and 1930 in the United Kingdom and Sweden [33]. Better childhood living conditions during this period was suggested as the reason. Thereafter, an inverse relationship between blood pressure and birth weight in the adult population born in 1946 in the United Kingdom was found [34]. In 1986, a strong correlation between geographical distribution of mortality rates from stroke and cardiovascular diseases between 1968 and 1978 and neonatal mortality in 1921–1925 was found by Barker *et al.* It was concluded that environmental factors, particularly nutrition during pregnancy and early life influence the risk of chronic diseases in later life. Based on these observations, the “early” or briefly “fetal” origins of adult disease (FOAD) hypothesis was proposed by Barker. According to FOAD, adulthood hypertension, insulin resistance and dyslipidemia occur due to adverse intra-uterine conditions and low birth weight [35,36]. Hence, it has been suggested that the metabolic syndrome be renamed “the small baby syndrome” [37]. This theory is based on “developmental plasticity”: There is a specific developmental period that an organism is responding to environmental factors by producing permanent changes in organs and/or regulatory systems as an adaptation process. The purpose of this adaptation process is increasing fetal survival rate. However, the evolutionary advantage of this adaptation process alters the phenotype of offspring and may increase risk of chronic diseases in later life. Thereafter, several hypotheses have been proposed to explain how environmental factors influence the developmental pattern in pre- and early post-natal periods that persists in later life. “Thrifty phenotype”, “fetal salvage”, “fetal insulin”, “catch up growth” and finally “predictive adaptive response” hypotheses have been proposed to explain fetal and early programming of adult disease.

“Thrifty phenotype hypothesis” proposes that in response to a suboptimal fetal environment, metabolic adaptations occur to maximize chances of surviving in conditions of post-natal food deprivation [38]. These adaptations can be beneficial if the poor conditions are continued, however, if the postnatal environment consists of a plentiful food supply, the risk of developing characteristics of the metabolic syndrome will be increased [39,40]. This interaction between *in utero* and post-natal environment has been captured in the Predictive Adaptive Response (PAR) hypothesis. According to the PAR hypothesis, offspring received a weaning diet similar to their mothers’ diet will have a more proper adaptation in response to environmental factors compared with those that received a different diet from their mothers’ diet [40]. Third, the catch-up growth hypothesis recognizes that there are interactions between early development and later nutritional exposure. Those that are growth restricted in the pre-natal period show a rapid post-natal increased adiposity in childhood and later adult life, associated with insulin resistance [41,42,43]. Fourth, the fetal insulin hypothesis suggests that both low birth weight and insulin resistance may be mediated by the same inheritable genes [44] because insulin plays an important role in fetal development [45]. Similarly, the “fetal salvage” hypothesis proposes an adverse intrauterine environment influence programming of endocrine pathways, leading to permanent metabolic changes. Finally, epigenetic regulation through the mechanisms of DNA methylation, histone modification, and microRNAs is crucial to the development and differentiation of cells as well as offering phenotypic plasticity in response to environment situations. Early nutrition can permanently influence the metabolism of offspring by influencing gene expression. Maternal protein restriction and caloric restriction during pregnancy can influence transcriptional factors involved in energy homeostasis, skeletal muscle development, and cellular memory [46]. Additionally, the time, the intensity, and duration of nutritional environmental assaults may induce different epigenetic alternations in a tissue dependent manner. For example, one of the first studies to confirm nutrition imbalance during intrauterine development induce epigenetic modifications demonstrated that feeding low protein diets to pregnant rats resulted in global DNA hypermethylation in livers of offspring. More recent studies have confirmed that maternal low protein gestational diets result in locus specific changes in DNA methylation that remain stable until adulthood. Conclusively, there may now be sufficient evident to support that maternal low protein gestational diets induce permanent alterations in gene expression through epigenetic modification [47].

## 3. Maternal Protein Intake and Fetal Programming

The role of protein content and source in maternal diets during pregnancy and post-natally on the risk of the development of characteristics of the metabolic syndrome in offspring has been investigated. Both protein content and source in maternal diets influence phenotype of offspring [12,14,15,16,17,23,24,25] and is discussed in the following.

### 3.1. Regulation of Food Intake, Body Weight and Composition

Birth weight was of interest of many studies as an indicator of fetal development and low birth weight has been proposed as a factor leading to higher risk of chronic diseases in later life. Barker *et al.*, for the first time, found an inverse relationship between birth weight and blood pressure in children at 10 years of age. Barker hypothesized that hypertension was due to an adverse fetal environment that increased the risk of cardiovascular disease in later life [48]. Low birth weight (5.5 pounds or 2.5 kg or less) is an indicator of malnutrition during pregnancy and serves as a crude indicator of slow fetal growth [49]. Low birth weight is associated with a range of metabolic diseases in adulthood, including type 2 diabetes, hypertension, and dyslipidemia [50,51]. However, whether catch-up growth followed by a low birth weight has a negative impact on health in later life is still unclear. In some studies rapid weight gain in the early postnatal period has adverse consequences for later health [52,53,54,55] while some others reported that low weight at one year of age or low weight gain in the first year of life can increase the risk of developing metabolic and cardiovascular disease later in life independently [56,57,58,59,60,61]. Birth weight also was inversely associated with truncal/peripheral fat ratio but not with relative body fat at 6 months [62]. However, in some other studies, altered maternal diet had negative health outcomes in offspring independent of birth weight [7,23,24,25]. Moreover, intrauterine growth rate and restriction are also determining factors and some babies born with a low birth weight are constitutionally small.

Protein content (low and high protein diets) and also protein source in maternal diets influenced body weight and body composition of offspring [23,24,61]. Both low and high protein maternal diets have detrimental effects on body weight and body composition of offspring.

#### 3.1.1. Human Studies

In human, childhood obesity can occur in various pathways through developmental origins of health and disease effects. For example, both maternal obesity [63] and starvation during pregnancy starvation *in utero* as occurred to fetuses exposed during the Dutch Hunger Winter increased risk of childhood obesity [64]. An association between low protein intake during pregnancy and lower placental weight and birth weight was observed in offspring of Caucasian women in Adelaide, Australia [65] and in Southampton, UK [66]. Similarly, offspring born to women on low protein diets due to restriction of milk during pregnancy (<250 mL/day) had significantly lower birth weight compared with women who consumed more than 250 mL/day milk [67]. Surprisingly, a high protein liquid supplement containing 40 g protein added to the diets of black women during pregnancy also resulted in lower birth weight in offspring compared to those born to mothers fed a low protein supplement (6 g as 7.5% energy) beverage [68]. The underlying mechanism for the latter effect is unclear at present but might be due to changes in placenta by increased number of villous capillaries relative to control and it might reflect increased metabolic activity and cellular proliferation in placenta secondary to the high density protein supplement [68]. However, authors indicated that there was no evidence of any placental change associated with the highly significant depressed birth weight among preterm deliveries in the supplement group.

In another study, the effect of maternal macronutrient and energy intake on food intake of offspring at 10 years was investigated (*n* = 5717 mother-child pairs prenatally and 5593 mother-child pairs postnatally). Protein and fat content of the maternal diet fed during gestation, when adjusted for energy intake, and carbohydrate had a positive association to dietary nutrient intakes by the child. A stronger relationship for protein and fat was observed in maternal prenatal-offspring intakes compared to protein and fat maternal postnatal-offspring intakes. Moreover, greater maternal energy intake was associated with greater adiposity in children but just after adjustment for energy intake. However, no effect of maternal prenatal macronutrient intake on offspring adiposity was observed. In general, prenatal maternal macronutrient intake had stronger association with offspring intake than postnatal and also paternal macronutrient intake [69].

Protein:carbohydrate:fat ratios may also play a role in fetal body composition and abdominal adiposity. Prospective data from 179 Australian women with singleton pregnancies from the Women and Their Children’s Health Study revealed that fetal body composition may be modifiable via nutritional intervention in the mother and may play an important role in the risk of future chronic disease in the offspring. Results from this study suggest that maternal nutrition during pregnancy cannot only lead to variations in the ratio of fetal fat to lean body mass but also fat site deposition areas. Researchers observed a positive relationship between fetal abdominal visceral adiposity and maternal protein:carbohydrate ratio, the development of fetal midthigh fat was driven by maternal diets containing intermediate protein, and an anti-obesogenic effect on offspring who had maternal PUFAs added to the diet. These observations suggest that there may be an ideal protein:carbohydrate ratio as well as an appropriate target macronutrient profile to optimize fetal body composition as well long term health outcomes [61].

Similarly, prospective data from 965 Danish pregnant women (1988–1989) with normal BMI (22.1 ± 3.3) revealed that substitution of carbohydrates for an animal protein source (mainly protein from meat sources) increased risk of BMI > 25 in both male and female offspring. However, lack of information on post-natal exposure in this study and also evaluating meat as primary source of animal protein are limiting factors for this study [70]. Moreover, the lack of information about the fat content of meat consumed during pregnancy as potential confounders is another limiting factor for this study*.*

#### 3.1.2. Animal Studies

Although both maternal low and high protein diets have been reported to increase body weight of rat offspring, their effect on birth weight is not consistent. Where low protein diets during pregnancy have led to low birth weight in most studies, high protein diets have been reported to result in lower, no effect or higher birth weight [71].

*Low protein diet:* A low protein content of the diet significantly influences body composition in rats. A low protein diet during pregnancy increased body weight and absolute weight of brown adipose tissue [72]. Brown adipose tissue has a significant role in the regulation of energy balance because it is a site of facultative thermogenesis that is mediated by uncoupling protein 1 (UCP1), a mitochondrial inner membrane protein [73]. In another study, when protein restriction was extended throughout gestation and immediately after birth, a decline in skeletal muscle mass was observed [74]. Moreover, gestational low protein diet resulted in greater adipose tissue during catch up growth, increased insulin-like growth factor 2 (IGF2) mRNA expression, and increased IGF2/H19 locus imprinting control region (ICR) differentially methylated region (DMR) DNA methylation in Sprague Dawley rat offspring, suggesting that maternal low protein diets influence adipose tissue catch up growth *in utero* by modification of the IGF2/H19 genome methylation [75].

More recently, the interaction between methyl donors (methionine, folic acid, betaine and choline) and protein content in maternal diet during the preconceptual (from 21 to 28 days before mating), pregnancy and lactation periods has also been investigated. A maternal diet low in protein and supplemented with methyl donors (folic acid: 15 mg/kg diet; methionine: 12 g/kg diet; betaine: 15 g/kg diet; choline: 15 g/kg diet) had no effect on food intake but did impair post-natal growth in both genders and also long-term weight gain in male offspring of rats only. Offspring born to dams fed a low protein diet with supplemented methyl donors and those born to dams fed a low protein diet had 45% and 21% less weight gain than control group respectively. Interestingly, rats born to dams fed a low protein diet that was supplemented with methyl donors gained less weight in response to a hypercaloric diet compared with offspring born to the control group. Moreover, leptin levels in plasma was 5 times lower in females born to dams fed low protein, methyl donor supplemented diet compared with control. However, this observation was not supported by the results from leptin gene expression which was only slightly lower in females born to dams fed low protein, methyl donor supplemented diet compared with control [76].

Furthermore, due to the important role of the gut in nutrient absorption, Pinheiro *et al.* investigated the role of a maternal hypoproteic diet on gene expression and the immunolocalization of the enterocyte transport proteins SGLT1, GLUT 2, and PEPT1 in 3- and 16-week old rat offspring. Staining intensity scores showed that the duodenum villi of gestational protein restricted rats 16 weeks of age had more intense immunoreactivity to SGLT1, GLUT2, and PEPT1 in the apical membrane of enterocytes when compared with controls. Additionally, the enterocyte proliferation of all intestinal segments was higher in animals born to protein restricted dams, especially in the jejunum of young rats. mRNA expression of SGLT1 and PEPT1 were also higher in the duodenum of intrauterine protein restricted offspring; yet mRNA expression of GLUT2 was not affected when compared with controls. These results suggest that poor nutrition in intrauterine life as well as during periconception produce adaptive responses that allow the fetus to maximize nutrient uptake and maintenance, supporting the “thrifty-gene hypothesis” [77].

The effect of maternal protein restriction during pregnancy and lactation on fiber-type composition of skeletal muscle in rats was studied by da Silva *et al.* Rats fed a normal protein diet (17% protein) exhibited enhanced density of type II fibers along with a decreased rate of fatty acid oxidation and glycolysis in the soleus but not the extensor digitorum longus (EDL) in the lower leg compared with rats that were fed a low protein diet (7%). Authors suggested that protein restriction alters the structure and enzymatic properties of skeletal muscle with a potential role in the development of obesity and its comorbidities as fiber-type composition of skeletal muscle is associated with obesity and insulin resistance [78].

*High protein diet:* A low-carbohydrate (CHO) (16.5% of total calories), high protein (26.9% of total calories), high-unsaturated-fat diet (HFP diet) fed before and during gestation and lactation resulted in higher birth weight in mice [19].

*High vs. low protein diet:* Limited and/or excess protein intake of pregnant gilts altered their offspring’s body weight and composition similar to the results seen in studies with pregnant dams. Both maternal high protein (30% *w*/*w*) and low protein diets (6.5% *w*/*w*) caused a reduction in offspring birth weight by 10% [51]. Body weight and hot carcass weight at 1 day of age were significantly lower in piglets born to mothers fed a low protein diet, but not in piglets born to dams fed a high protein diet when compared to controls (12.1% *w*/*w*) [79]. In addition, the sum of all internal organ weights tended12 to be reduced by 11% in offspring in response to a maternal low protein diet. Weight of skeletal muscle tissue was also lower in low protein offspring compared to those born to mothers fed a high protein diet. Authors suggested that the formation of myofibers during prenatal myogenesis is significantly impaired in response to a low protein diet. Skeletal muscle has a major role in regulating metabolic homeostasis. Therefore, the reduction in biochemical and nucleic activity as well as limited formation and size of skeletal muscle in low protein piglets may lead to metabolic dysfunction and adult onset of chronic disease [79].

*Protein source:* Protein source in a nutritionally adequate maternal diet influenced body weight and composition in offspring [23,24,25]. In offspring of dams fed the soy protein-based diet body weight was 9.4% higher compared with offspring of dams fed a casein-based diet at week 14 post-weaning. Failure of devazepide (a cholecystokinin-A (CCK-A) receptor blocker) to diminish food intake suppression induced by protein preloads in offspring born to dams fed soy protein-based diet was an indicator of altered food intake regulatory system. Moreover, hypothalamic gene expression of agouti related protein (AgRP) in offspring born to dams on the soy protein-based diet was higher at weaning. Food intake was also higher in those offspring in entire post-weaning period [23]. Another indicator of altered food intake regulatory system in response to maternal diet was changes in intake regulatory hormones in offspring: At weaning plasma insulin and at week 15 post-weaning insulin, ghrelin, and GLP-1 were higher in offspring born to dams fed the soy protein-based diet [24]. Authors suggested that extending the diet from gestation alone to throughout gestation and lactation exaggerated the adverse effects of the soy protein-based diet. However, post-weaning diet consumed by offspring had only a mild effect on phenotype of offspring.

Conversely, Bautista *et al.* suggest that the source of protein consumed post-weaning has a tremendous effect on dietary manipulated pregnant and lactating dam adult offspring. Authors stated that intrauterine and postnatal diet mismatch leads to maladaptive phenotypes during adulthood; however, dietary intervention and protein source can improve some specific outcomes that manifest in adulthood resulting from maternal protein restriction during gestation and lactation. In Bautista’s *et al.* study offspring of protein restricted dams were fed either a casein based chow after weaning or a corn and soybean protein based chow (Chow Purina 5001). Protein restricted offspring given the casein based chow had a higher body weight, a higher percent of carcass fat, and a higher amount of liver fat. Protein restricted offspring that were switched to the corn and soybean protein based chow showed a decrease in adult body weight, a lower body fat percentage, and exhibited a loss in liver fat after the dietary intervention. It suggests that dietary intervention in adult life can improve some of the specific outcomes due to maternal protein restriction; hence in order to avoid the deleterious effects of programming, one should adopt, as early as possible a healthy lifestyle that not only maintain but improve homeostatic parameters associated with prenatal and neonatal maternal protein restriction [80].

This controversy can be explained by the fact that Jahan-Mihan *et al.* compared maternal (during gestation and lactation) and weaning casein- and soy protein-based AIN-93 G lab powdered diets which are nutritionally balance and adequate [24,25]. However, Bautista’s *et al.* used casein as source of protein in both restricted and normal protein maternal diet groups in the form of biscuit while offspring received an adequate casein-based diet until day 70 post-weaning following by either a casein-based or a commercial diet (Chow Purina 5001, Mexico City, Mexico) with combined corn and soy bean proteins as source of protein with an unexplained ratio. Therefore, it is difficult to interpret the results of this study based on the source of protein because the source of protein in maternal diet was the same (casein) in 2 groups and offspring received only casein diet during lactation and early post-weaning period (until day 70 post-weaning) following by either a casein-based or a commercial diet (usually with more variation in ingredients compared with a lab diet) with a mixed sources of proteins. Moreover, this study was on female offspring while Jahan-Mihan’s study was on male offspring. In general, the results from Bautista *et al.* [80] study support the significance of the weaning diet when maternal diet is either protein restricted or sufficient while Jahan-Mihan’s study support the notion that both gestation and lactation periods are important and lactation can amplify the effect of gestational diet.

### 3.2. Regulation of Blood Glucose

The effect of both low- and high-protein diets during pregnancy and early life on the development of glucose intolerance and diabetes in offspring has been studied extensively in animals. However, no effect of low protein diet on glucose metabolism in dams during gestation has been reported.

*Low protein diet:* In one study offspring born to low protein (8%–10% *w*/*w*) fed dams have enhanced glucose tolerance and increased insulin sensitivity during early life [17,81] in muscle [82] and adipose [83] tissues. However, impaired glucose tolerance occurs by 15 months of age [84] and diabetes at 17 months of age [85]. Normal fasting plasma glucose and insulin levels in young offspring born to the dams fed a low-protein gestational diet is reported by some studies while impaired glucose tolerance in adult female offspring due to lower insulin response to an oral glucose preload is reported by others [86].

Massive β-cell remodeling takes place in the fetal sheep during late gestation. Therefore, the effect of diet fed during late gestation and post-natal periods on glucose metabolism was examined by Kongsted *et al.* [87] Offspring born to twin-pregnant sheep fed a low energy, low protein diet (50% of normal diet) in late gestation developed insulin resistance compared with those fed a normal diet. Insulin AUC was significantly higher in lambs born to mothers fed a low protein low calorie diet in late gestation compared with those born to mothers fed a normal diet regardless of lambs’ diet. Interestingly, the lambs’ post-natal high CHO, high fat diet exaggerated the adverse effect of low protein low calorie maternal diet fed during late gestation as evidenced by higher glucose AUC. Authors suggested that dietary alterations and weight reduction later in life can prevent the adverse outcomes of the early post-natal over-nutrition but not the adverse effects of late gestational malnutrition [87]. However, it is particularly important to take into account that these results can be interpreted as outcome of interactive effect of protein and energy restriction and they cannot be attributed to either alone.

The role of low protein diet (10%) fed during pregnancy in aging-related development of dysfunction of insulin metabolism has also been studied. A low protein maternal diet fed during pregnancy had a negative impact on aging-associated glucose-stimulated insulin secretion loss in rats; therefore, authors propose that developmental programming is a major factor in aging-related development of dysfunction of insulin metabolism. Results from such studies show that at postnatal day 450, insulin secretion to various glucose concentrations (5, 7.5, 11 and 22 mM) were significantly reduced in offspring born to dams fed a low protein diet compared with those born to dams fed a normal protein diet and this effect was independent from offspring’s diet [88].

*High protein diet:* In a study, pregnant gilts were fed either high protein/low carbohydrate (HP-LC; 30% protein, 39% carbohydrates *w*/*w*) or a low protein/high carbohydrate diet (LP-HC; 6.5% protein, 68% carbohydrates *w*/*w*) or a standard diet (ST; 12.1% protein, 60% carbohydrates *w*/*w*). HP-LC fetuses had intrauterine growth restriction (IUGR) and asymmetrical growth of fetuses within the same litter [89]. The role of altered maternal glucose metabolism in growth restriction was studied by examining glucose, fructose, lactate, and inositol concentrations in the fetal arterial and venous umbilical cord [89]. Fetuses of dams’ sows fed a HP-LC diet had 50% less glucose concentration in the umbilical vessels compared with maternal concentrations. Moreover, umbilical lactate levels were twice as high of those found in dams. Lower umbilical arterial lactate levels in fetuses of LP-HC fed sows was observed compared with the controls. Inositol concentrations in umbilical plasma in HP-LC fetuses were also higher when compared with fetuses of ST. Moreover, higher concentration of inositol was found in the small fetuses and lower concentration was found in the large fetuses. It may suggest that generation of higher amounts of inositol in smaller fetuses counteract an insufficient nutrient supply. Further research is needed to determine if high inositol levels accompany IUGR [90].

### 3.3. Regulation of Liver Triglyceride and Cholesterol Metabolism

*High vs. low protein diet:* Preliminary investigations in porcine model indicate that high protein diets fed during pregnancy affect the hepatic expression profiles in a short term as well as a long term in offspring fed high protein diet. Moreover, no significant alteration in regulation of metabolic pathways were observed in porcine fetal livers when a high protein diet fed during pregnancy [89].

*High vs. Adequate vs. Low protein diet:* Interestingly, the lack of metabolic insult to the liver within the porcine model may be explained by the “hierarchical order” of gene expression in tissue modulation as a result of gestational dietary offense. To investigate this new phenomenon, Oster *et al.* evaluated hepatic and muscular gene expression profiles of the progeny of sow fed either an isoenergetic maternal low protein/high carbohydrate (LP) diet, a high protein/low carbohydrate (HP) diet, or an adequate protein (AP) diet throughout pregnancy. Transcriptional comparisons of hepatic transcripts among dietary groups revealed no differences between AP and HP offspring, but three difference within the probe-sets of LP and HP offspring; however, when comparing skeletal muscle transcript’s, researchers discovered 180 difference between AP and HP offspring, 177 difference between LP and AP offspring, and 855 transcriptional differences between LP and HP offspring. In particular, researchers found that expression of genes related to oxidative phosphorylation and fatty acid metabolism were increased in LP compared with HP skeletal muscle. Based upon these transcriptional comparisons between differing tissue and dietary insults, researchers within this study concluded that liver tissue may be highly resilient to nutritional alternations whereas, skeletal muscle tissue may be less resilient to nutritional insults and is more likely to show more transcriptional differences than the liver when the gestational diet has been altered [91]. Perhaps in the event of nutritional inadequacy during fetal development, differing tissues do not experience a common transcriptional response or follow a universal target pathway in epigenetic variations. There is a distinct possibility that higher functioning organs may be genetically “protected” from nutritional inadequacies as evidenced by Oster’s preliminary results; however, more research is needed in addressing the concept of genetic and physiological plasticity differences between tissues.

### 3.4. Immunity

*High vs. low protein diet:* To date few studies in rodents have examined the effects of altered protein nutrition throughout pregnancy on offspring immunity. Preliminary studies have shown that both low (4%) and high (20%) dietary protein levels during gestation led to alterations in plasma protein, albumin, and gamma-globulin levels of neonate rats when compared with neonates exposed to a 10% protein control diet. In addition, impaired thymocyte proliferation at birth and thymic and spleen lymphocyte proliferation at weaning were altered in the offspring of dams fed a moderate protein restricted diet. These findings are especially relevant because in addition to low birth weight, altered maternal protein diets may affect immune reactivity and physiological adaptive responses of offspring to immune challenges later in life [92].

Tuchscherer *et al.* examined the effects of a low (6.5%) and a high (30%) protein:carbohydrate ratios in the diet of sows on the immune system of their offspring at significant ages. Findings suggest that both low and high protein:carbohydrate ratio throughout pregnancy affected baseline immune parameters in neonatal offspring and also modify immune responses to stress later in life. Significant differences in the concentrations of immunoglobulins between diet groups were seen on the first day of life: IgG and IgM levels in HP piglets were lower when compared to AP piglets. Additionally IgA levels were lower in both LP and HP piglets when compared to AP piglet offspring. Negative correlations between the cortisol levels of sows and IgA levels of piglets were also seen in both the LP and HP groups but not in AP groups. Of note, cortisol levels of sows and the IgM concentrations in piglets were negatively correlated in the LP group, but not in the AP and HP groups [92].

At weaning, LP piglets displayed a significantly higher cortisol level, suggesting that gestation protein restriction may alter hypothalamic-pituitary-adrenal axis regulation to stressful or challenging situations; hence the immunity of LP neonatal pigs in response to the stress may be compromised. Furthermore, the percentage of CD4+ cells and the CD4+/CD8+ ratios were significantly increased after the stress of weaning in HP piglets only. Chronic stress in sheep and humans has also been shown to increase the CD4+/CD8+ ratio.

Overall, maternal protein malnutrition during pregnancy can alter immune responses in offspring, specifically during times of challenge or stress. Both low and high protein:carbohydrate ratios in gestational diets moderately decrease humoral immunity in neonatal offspring, and modify adaptive coping responses to stress later in life [92]. However, the mechanisms are still elusive.

### 3.5. Regulation of Blood Pressure

*Low protein diet:* In humans, a low protein/carbohydrate ratio in the diets available during the Dutch famine had a stronger association than birth weight with high blood pressure in later life [93]. However, the results from animal studies are contradictory. Langley and Jackson (1994) reported that feeding low-protein diets (6%–12% of total calories) to rats during pregnancy resulted in an increase in blood pressure [94] whilst, Lucas (1996) reported that low protein diets (8% of total calories) during pregnancy had no effect on blood pressure in the offspring [95]. Similarly, two widely used low protein, casein diet formulations (9% of total calories), the University of Southampton diet and that produced by the Hope Farm Company in the Netherlands have been applied to examine the effect of low protein diet on programming of blood pressure but gave contradictory results. When the Southampton diet was given to the dams, higher systolic blood pressure was found in the offspring at 4 week of age [96,97]. However, the Hope Farm diet, resulted in normotensive, insulin-resistant offspring [82,95,98]. Because these diets differ in sources of carbohydrate (starch, glucose and sucrose), fat source and content, and choline and methionine content, it is clear that the effect of the low protein diet can be modulated by other characteristics of the diet. Unfortunately they have not been identified. Extending maternal low protein diets to the postnatal period results in a more robust effect in increasing blood pressure [99]. Dams were fed a low-protein diet (8% of total calories) throughout gestation and lactation. The same diet was given to the offspring until 70 days of age and it was followed by a highly palatable cafeteria-style diet. Low protein diet during lactation significantly increased blood pressures, as it did in the cafeteria-fed rats. Authors suggested that early protein restriction and later obesity are independent risk factors for the development of hypertension [99].

The mechanisms by which a low protein maternal diet alters blood pressure in the offspring are unknown. Increased peripheral resistance has been suggested due to lower pulse rate in the absence of cardiac hypertrophy, indicating that cardiac output is not elevated [100]. However, a low protein gestational diet has also been reported to lower heart size with no difference in pulse rate in rat offspring [101].

The disruptions in normal circadian rhythm could be another possible mechanism for altered blood pressure in offspring born to dams fed low protein diet. A maternal low protein diet led to the loss of circadian rhythms of the blood pressure and heart rate in mice offspring born to dams. At 9 months of age, low protein offspring lost the difference in blood pressure between day and night which normally occurs. It is particularly important because many homeostatic functions such a temperature, eating, blood pressure and heart rate are influenced by the circadian clock. Therefore, disruption of this rhythmic mechanism may correlate with a higher risk for hypertension and cardiovascular complications [102].

Moreover, any alteration in mechanisms controlling breathing can increase risk of the development of hypertension. In one study, male rat offspring born to mothers fed low protein diet had higher respiration frequency and volumetric flow rate of air and no change in tidal volume at 30 days of age. At 90 days of age, respiration frequency was still higher but volumetric flow rate of air and tidal volume had returned to normal levels when compared with control. These findings suggest that low protein maternal diet fed during prenatal development differentially alters the ventilatory responses and this effect is age dependent. Therefore, it can be suggested that there may be a relationship between early changes in ventilation due to maternal protein restriction and the onset of high blood pressure in adulthood [103].

*High protein diet:* Although the role of low protein diets during pregnancy on blood pressure of the offspring has been investigated widely, the role of high protein diets has received less study. Young adult humans had an increased systolic blood pressure at ages 27–30 year when born to women consuming a high-meat low carbohydrate diet during pregnancy (0.45 kg meat/day) [104]. In Wistar rats, when were fed either a normal (20% of total calories) or high protein (40% of total calories) diet throughout pregnancy and lactation, blood pressure was higher at week 4 of age and persisted throughout the study in male offspring born to high protein fed dams [20].

In summary, there is substantial evidence that both protein content and source in the dams’ diet affect blood pressure in the offspring. However, the mechanisms behind these responses have received little examination. Thus, in the following a background is given first on proposed mechanisms of fetal programming and then followed by an exploration of how protein content and composition in diets may affect fetal programming.

## 4. Mechanisms of Fetal Programming

### 4.1. General Mechanisms

The underlying mechanisms by which fetal programming is influenced by nutrition are not completely understood. However, both clinical and experimental studies demonstrated that hormones are environment-dependent organizers of the neuroendocrine system, which ultimately regulates all fundamental processes of life. Therefore, non-physiological concentrations of hormones due to altered intra-uterine and/or early post-natal environment can act as “endogenous functional teratogens” by malprogramming of the neuroendocrine-immune system leading to developmental disorders and chronic diseases in later life [105].

Animal studies support a role for cortisol, leptin, insulin and ghrelin in intra-uterine and early post-natal development during malnutrition during pregnancy plus lactation. While these hormones have been the focus of many investigators there is a high likelihood that many others are affected by the maternal diet and in part on the fetus.

### 4.2. Corticosteroids

Administration of glucocorticoids to pregnant animals and humans leads to intrauterine growth restriction (IUGR) [106] which has long-term clinical consequences induced by fetal programming [107]. During intrauterine undernutrition, the fetus is exposed to higher levels of glucocorticoids thus leading to the suggestion that it is a significant factor influencing the offspring [108,109].

Excessive glucocorticoids during the third semester of pregnancy (3rd week) resulted in hyperglycemia, glucose intolerance, and hyperinsulinemia in adult life in rats [110]. Elevated blood pressure in adults born of low birth weights have been correlated with greater corticosterone concentrations, especially in obese individuals [108]. Maternal low protein diet (8% cal) has also been shown to significantly increase the plasma corticosterone levels in offspring and this effect was amplified by an obesogenic post weaning diet [102].

Up regulation of the gluconeogenic enzymes Glucose-6-phosphatase, catalytic subunit (G6PC) and Phosphoenolpyruvate carboxykinase 1 (PCK1) that ultimately stimulate gluconeogenesis earlier in pregnancy was observed in IUGR piglet fetuses as a result of malnutrition combined with stress hormones including glucocorticoids and catecholamines [90]. Alterations in the expression of G6PC have led to disorders and diseases involving glycogen storage. Moreover, as PCK1 is a key regulator in gluconeogenesis, its causal role in the development of diabetes has been studied in type 2 diabetic patients. In offspring born to sows fed a high protein/low carbohydrate gestational diet, increased hepatic transcript concentrations of PCK1 were observed when compared with controls. However, in offspring of sows fed a low protein/high carbohydrate gestational diet G6PC levels were increased compared with controls [90].

### 4.3. Insulin

Insulin plays an important role in fetal growth [45] and elevated insulin is associated with development of obesity and diabetes [111]. A positive correlation between the level of amniotic insulin or perinatal hyperinsulinemia and the increase in body weight and the risk of impaired glucose tolerance in later life in offspring of diabetic mothers has been reported [111,112,113]. The risk of obesity and glucose intolerance in later life is higher in offspring born to mothers with maternal or, gestational diabetes and or even with a mild glucose intolerance [114,115].

Insulin may malorganize neuroendocrine systems by effects on hypothalamic controllers [116]. The hypothalamus is particularly sensitive to levels of circulatory hormones during the prenatal period [116]. Increased insulin concentration within the immature hypothalamus leads to permanent dysplasia of central nervous nuclei regulating metabolism and body weight in the ventromedial hypothalamic nucleus (VMN) [115,116,117,118,119]. Moreover, hypothalamic resistance to satiety signals insulin and leptin is associated with a life-long increase in activity and number of orexigenic peptides galanine and neuropeptide Y in rats [120,121]. Neonatal insulin treatment induces morphological alterations in hypothalamic structures that leads to the development of obesity and adult hyperinsulinemia in rats [122].

### 4.4. Leptin

Leptin is an adipose-derived, anorexigenic hormone secreted in proportion to fat mass [123]. Leptin limits the expression of both Neuropeptide Y (NPY) and agouti-related protein within hypothalamic arcuate nucleus [121] to control weight gain, feeding behavior, and metabolism [124]. Data from human studies indicates that low maternal concentrations of plasma leptin increases risk of obesity in offspring [125]. Individuals with low birth weight tend to have higher leptin concentrations in adulthood [126].

Leptin plays a role in early post-natal development of hypothalamic circuitry [127]. Thus, it has been suggested that an abnormal profile of post-natal leptin concentrations are causative in the later development of an obesity-prone phenotype [128]. Offspring of rats injected subcutaneously with leptin during the first ten days of life have increased food intake, body weight and reduced responsiveness to leptin in later life associated with lower levels of ObRb (a leptin receptor b isoform) expression in the hypothalamus [129].

### 4.5. Ghrelin

Ghrelin is an orexigenic peptide synthesized and secreted from the fundic region of the stomach and also in other tissues and other parts of gastro-intestinal tract [130].

Although ghrelin is primarily secreted by the stomach and upper intestine, it is also expressed in human placenta. The highest levels of ghrelin are detected at mid gestation [131] suggesting that ghrelin plays an important role in pregnancy with a role in the pregnancy-related maternal weight gain [132] and fetal birth weight in rats [133]. Plasma ghrelin concentration is influenced by diabetes mellitus (DM) during pregnancy.

In human, in a population of pregnant women, ghrelin levels were lower in type 1 DM pregnancies at 20 and 30 weeks (*n* = 12, age 29.9 ± 4.7, BMI: 27 ± 3.1) compared with healthy pregnant women (*n* = 12, age 31 ± 5.5, BMI: 25.2 ± 3.7) [134]. In type 2 diabetic adult patients, decreased plasma concentrations of active ghrelin are significantly associated with abdominal adiposity, hyperinsulinemia, and insulin resistance [135]. Moreover, insulin resistance and hyperinsulinemia are inversely associated with ghrelin concentration in obese individuals [136]. Insulin and ghrelin expressions in plasma and pancreatic islets were influenced by long term high fat/high energy diets and also high protein diets, suggesting that ghrelin secreted by pancreatic islets may regulate insulin release and the self-replication of beta cells. However, further studies are needed to determine the physiological and pathophysiological roles for circulating ghrelin in the regulation of insulin release as well as the associations between pancreatic ghrelin and beta cells [137].

## 5. Proposed Mechanisms of Fetal Programming by Proteins

Although the role of protein contents of maternal diet in the development of chronic diseases in offspring provide evidence that they modify the development of somatic structure and phenotype of the offspring, physiological responses to dietary proteins are determined by not only the concentration but also by the physiologic characteristics of proteins arising from their amino acid composition, bioactive peptides and digestion kinetics. Thus, it can be predicted that nutritional adequacy of amino acids may not be the only characteristics of the maternal diet to impact on the offspring.

### 5.1. Amino Acid Composition

Amino acids, independent of the protein content of the maternal diet may influence the risk of development of chronic diseases in offspring by affecting gene expression presumably through one carbon pathways of epigenetic mechanisms. For example, low protein diets with similar protein content (8%–9% of total calorie) affect programming of blood pressure differently. The low protein Southampton diet results in higher systolic blood pressure in offspring [96,97], whereas the Hope Farm diet had no effect [82,96,98]. Increased maternal serum levels of homocysteine occur after only 4 days of feeding [138] and hyperhomocysteinemia is associated with alteration in gene methylation status in the liver of fetuses [15]. Moreover, hypomethylation of DNA can be related to elevated homocysteine (Hcy) concentration [139] that consequently alters organogenesis and in embryonic vasculogenesis by influencing its major events [140].

Adding glycine which reduces plasma homocysteine to the “Southampton” diet normalizes blood pressure. It may suggest that the methionine load is a contributor to the phenotype of the “Southampton” diet [100]. Supplementation of the low-protein diet with threonine during the initial phases of pregnancy has been reported to both decrease [138] and increase maternal concentrations of homocysteine [15]. Supplementation of a low-protein gestational diet with taurine (2.5%) restored normal insulin secretion [141]. Taurine is also involved in homocysteine metabolism and reduces the demand for cysteine.

Several studies have demonstrated that taurine supplementation has potential as a regulator of insulin secretion and promotes insulin sensitivity; moreover, taurine can ameliorate fructose-induced hyperglycemia, hypertension and hepatic steotosis in pregnant and non-pregnant rats. In investigations where rats were fed a high fructose gestational diet, taurine supplementation helped to reverse indices of hypoglycemia and elevated plasma β-hydroxybutyrate (BHB) concentrations in neonates. Specifically, in male offspring of fructose fed dams, taurine supplementation reversed fructose-induced hepatic Phosphoenolpyruvate carboxykinase (PEPCK), a potential modulator of adult onset dysregulated glucose metabolism. Neonatal hepatic pro-inflammatory cytokine expression was also down regulated following maternal taurine supplementation. Taurine supplementation in fructose fed dams also increased litter size and while also decreasing the amount of neonatal deaths. This effect may be due to taurine’s ability to decrease elevated levels of plasma BHB in neonates [142].

Additionally, taurine supplementation in pregnant rats fed a low protein diet has been shown to normalize pancreatic islet development and glucose and insulin homeostasis in offspring, with benefits extending into adulthood; however, taurine supplementation in controls led to an increased incidence of neonatal deaths. As the exact mechanisms of taurine toxicity are not known, the supplementation of taurine on adequate gestational diets needs further investigation [142].

Current research is also focusing on the role of amino acid metabolism in intestinal bacteria and its potential implications for mammalian reproduction. As the numbers of bacteria in the intestine exceed the numbers of host cells by as much as 10 fold, there seems to be emerging compelling evidence that indicates that amino acid metabolism within the gut may play a crucial role in both female and male reproduction. The underlying mechanisms are unknown but may be related to: (1) the regulation of the digestion and absorption of specific amino acids vital to embryonic growth; (2) availability of dietary amino acids for use within the reproductive organs; (3) changes in gut microbial and amino acids metabolism associated with conception and pregnancy; and (4) changes in the production of reproduction related amino acid metabolites such as nitric oxide (NO), polyamines, and glutathione. Physiological levels of microbial changes and their by-products may influence cellular signaling pathways and reproduction throughout the lifecycle from the formation of gametes all the way to pregnancy and lactation. Hence, adequate dietary intakes of not only proteins but also both nutritionally essential amino acids and nutritionally nonessential amino acids are required for not only optimal intestinal health, but also reproductive health [143].

### 5.2. Bioactive Peptides (BAPs)

Although many physiological functions of proteins are attributed to BAPs, their role in the development of regulatory systems is unknown. I human, BAPs have been detected in the plasma of pregnant and lactating women [144,145] but whether BAPs cross the placenta and influence fetal development directly or alter fetal development indirectly by influencing maternal metabolism has not been shown.

BAPs have physiological functions in both human and animals [146,147]. For example, BAPs with angiotensin converting enzyme (ACE) inhibitory activity lower blood pressure in experimental animals. Casokinins originate from all major subunits of casein, α_s1_-, β-, and α-caseins [148] and their activities are much higher than those from soy protein. Vasoactive peptide [149] originates from α_s1_-casein fragment 25–27 and has IC_50_ of 2 μM [147]. Moreover, β-casomorphins, one of the major groups of BAPs abundant in casein [150,151] affect food intake regulation [152], gastro-intestinal motility [153], and plasma insulin concentration [154]. β-casomorphins interact with gastric opioid receptors slowing gastrointestinal motility [153,155]. Moreover, casomorphins decrease insulin secretion in the absence of elevated glucose levels but have no effect on glucose stimulation of insulin [156]. In addition, it has been proposed that BAP released from ingested proteins and delivered via portal vein may reduce first pass hepatic insulin extraction and lead to improved insulin sensitivity without resulting in increased insulin secretion [157]. However, these studies focus on adults and whether BAPs have any physiological property during development is still elusive and need further study.

### 5.3. Digestion Kinetics

The rate of digestion of the proteins and the resulting hormonal responses in the dams and peak amino acid concentrations in the fetus may also influence the development of regulatory systems. The digestion and absorption kinetics of dietary proteins influence catabolic and anabolic activities at the whole-body level [158] and in the liver [159] and brain amino acid concentrations and neural activity [160,161]. Based on their rate of digestion and absorption, proteins can be classified as either “fast” or “slow” proteins [159,161]. For example, casein is as slow protein and whey and soy protein are fast proteins. Plasma concentrations of serine, tyrosine, valine, isoleucine, branched chain amino acids (BCAAs), lysine and total amino acids are higher, and arginine and tryptophan are lower after a casein meal compared with after a soy protein meal in humans [162]. In addition, because of the more rapid absorption of soy protein, a larger portion of the amino acids are degraded to urea, resulting in less protein synthesis than after consumption of casein [162]. Hormonal responses to these proteins are markedly different. For example, a higher concentration of plasma insulin is noted after whey protein consumption as compared with casein at 60 min after gestation [163]. However, to our knowledge, there is no study examining the role of proteins’ digestion kinetics on fetal programming.

### 5.4. Hormones

Hormones play a key role in *in utero* and post-natal development. Proteins in maternal diet may influence fetal development by their effects on hormonal responses in the mothers. The effects of low protein diet on corticosterone, insulin and leptin have been shown.

*Low Protein Diet:* In pregnant rats fed a low protein diet, a decrease in activity of 11 β-hydroxysteroid dehydrogenase (11 β-HSD) occurs [153]. 11 β-HSD metabolizes maternal glucocorticoid transport to the placenta. Therefore, a decreased activity of placental 11 β-HSD activity will increase fetal exposure to maternal cortisol [164]. The detrimental effects of excess exposure of the fetus to glucocorticoids on growth, blood pressure and glucose metabolism of the offspring are well-documented [106,108,109].

Insulin is also a factor because maternal low protein diets increase the risk of insulin resistance in male offspring at 20 weeks of age [165]. Higher concentrations of insulin within the immature hypothalamus in fetus result in permanent alterations in life-long dysplasia of central nervous nuclei regulating food intake and BW [116,166] and may decrease sensitivity of hypothalamus to insulin and leptin [117]. Furthermore, maternal low protein diet may influence plasma concentrations of Hcy throughout altered insulin metabolism in offspring of rats [15]. In addition, plasma insulin in the insulin resistance rat model correlates positively with plasma Hcy [167].

Gestational protein restriction also reduces plasma leptin concentrations in mothers but not in fetus in rats [168]. This has been associated with an impact on the development because exogenous administration of leptin in the week of pregnancy and throughout lactation resulted in male offspring that were less susceptible to the effect of protein deficiency. They were also more resistant to diet-induced weight gain, fat pad gain and insulin resistance when fed a high-fat diet [168].

Studies of multigenerational effects of prenatal manipulation in the F1 and F2 generations and beyond are limited. Hormone disruptions that cause epigenetic alterations may also play a role in the long term effects on progeny through multiple generations, not just first generation offspring of protein malnourished dams. Reyes-Castro *et al.* investigated the paternal line multigenerational passage of altered risk assessment behavior, as a result of blunted corticosterone levels, in second generation female offspring of low protein diet fed dams [169]. First generation males of dams fed low protein gestational diets had lower corticosterone levels which can result in risk taking behaviors. First generation female offspring of low protein fed dams presented with high corticosterone levels, which are associated with higher anxiety levels. Interestingly, second generation female offspring from the paternal line had the same low serum levels of corticosterone and exhibited similar risky behavior as their fathers. There are several proposed mechanisms by which sex-specific transmission of phenotypes can occur including alterations in metabolic environment (transmaternal inheritance of obesity), gene expression mediated by developmental and epigenetic pathways (transpaternal inheritance of low body weight), or both (impaired glucose tolerance) [170]. Authors suggest that epigenetic modifications through the paternal lineage are manifested more robustly in second generation females [169]. However, there is no evidence indicating epigenetic traits as possible mechanism in current study. Authors also suggested that protein restrictive diets can have impact on the hormone regulation of multiple generations [169]. This study supports the notion that the transmission of transgenerational outcomes can be specific to either maternal or paternal route and it can influence the phenotype of the second generation in a sex-dependent manner. In another study, post-natal protein restriction resulted in insulin resistance in male rats while females developed insulin sensitivity in second generation [171].

### 5.5. Hypothalamic Development and Food Intake Regulation

The characteristics of metabolic syndrome that appear in later life of offspring exposed to malnutrition in uteru suggest that they may be simply secondary to obesity which in turn may be a consequence of either increased food intake due to altered development of intake regulatory system in hypothalamus. Thus, factors affecting development of hypothalamus are reviewed here.

Components of the central neural network for regulating food intake are present before birth in rodents and higher-order mammals [172,173,174,175]. However, unlike human and sheep, the neuronal circuitry is not fully developed until 16 days after birth in rodents [172,176,177]. In the rat, at 14.5 days gestation, neuropeptide Y (NPY) neurons appear in the arcuate and dorsolateral hypothalamus first [175,178,179] following by a rapidly increased NPY mRNA expression between 2 and 15–16 days after birth. Ultimately it returns to adult levels at around 30 days of age [172]. Functional NPY receptors exist at early life. The observation that microinjection of NPY directly into the PVN at 2 days after birth motivates milk and water intake support the role of NPY at early life [180]. Moreover, vagal sensory information from the gut relating to gut fullness may play a significant role in regulation of food intake in the first week of life. It could be due to a relative dominance of NPY and α-MSH innervation of the paraventricular nucleus (PVN) by efferents from the brain stem instead of the arcuate nucleus during this period [172]. However, NPY/AgRP projections from the arcuate nucleus to the DMN are not complete until some 10–11 days after birth, and projections to the PVN do not fully develop until 15–16 days [172]. Peripheral leptin treatment at *day 10* after birth reduces NPY mRNA expression in the rostral arcuate nucleus. However, leptin treatment has a mild impact on food intake and it can be explained by lack of NPY projections in hypothalamus during the early postnatal period [181]. Pro-opiomelanocortin (POMC), AgRP, and MC4R mRNA are also all present in the hypothalamus in early postnatal period. 

The perinatal period is a critical time for the programming of postnatal appetite in rats [182]. Plagemann and colleagues reported that increased nutritional intake due to small litters induced hyperphagia and obesity combined with hyperleptinemia, hyperglycemia, hyperinsulinemia, and insulin resistance in rats that causes various alterations in hypothalamic structures, neuropeptide levels, neuronal activity and hormonal responsiveness [166,183,184]. It was associated with increased in NPY and galanin expression and decreased responsiveness to leptin, insulin and neuropeptides within neurons of the arcuate nucleus (ARC) and PVN [141,185,186]. Insulin treatment on a daily basis between 8 and 11 days after birth also increased body weight gain, impaired glucose tolerance, chronic hyperinsulinemia, and hypertension in later life. Moreover, it resulted in morphological alterations in hypothalamus that persist in adult life [187,188,189]. It supports the notion that perinatal hyperinsulinemia confers malformation of hypothalamic structures.

Low protein diet when fed during gestation and lactation induced hypoinsulinemia, normalized plasma leptin concentrations, an increased NPY levels in the arcuate nucleus, PVN and lateral hypothalamic area. However, it did not change NPY levels in the VMN [190]. Food intake of the offspring was not measured. It is consistent with our observation that offspring born to dams fed the soy protein-based diet had higher food intake after weaning, higher hypothalamic mRNA expressions of AgRP at weaning and relatively higher plasma concentrations of insulin in fetal period (day 20 gestation) compared with those from dams fed a casein-based diet [23].

Additionally, a maternal low protein diet fed during gestation and lactation impact hypothalamic mammalian target of rapamycin (mTOR) activation in adult rat offspring. mTOR is one of the regulators of feeding behavior and integrates neuro-endocrine signals with nutrient signals from gastro-intestinal tract in the hypothalamus. Therefore, mTOR is involved in systemic regulation of energy homeostasis. Any lateration in mTOR signaling due to protein restriction during gestation may play a role in the developmental programming of metabolic disorders [191].

Guzman-Quevedo *et al.* found that adult rats born to dams fed a low protein diet during gestation and lactation exhibit enhanced activation of hypothalamic mTOR in the fed state as well as impaired mTOR responses to fasting and re-feeding from one hypothalamic nucleus to another. Protein restricted adult rats exhibited a decrease in number of phosphorylated rpS6 and pmTOR immunostained cells in the VMH and ARC but increased numbers of pmTOR immunopositive cells in the PVN under ad libitum feeding conditions. In controls, however, the phosphorylation of rpS6 and mTOR in the VMH decreased with fasting, whereas in malnourished rat offspring, fasting decreased the phosphorylation of mTOR in the PVN of the hypothalamic nucleus. No differences in the number of POMC/pmTOR co-labelled cells were found between control and malnourished rats in the fed state and both groups exhibited a significant decrease in the activation of mTOR in POMC expressing neurons in response to fasting, suggesting that early protein restriction may not alter the nutrient sensing function of mTOR in POMC neurons [191].

### 5.6. Pancreatic Development and Metabolic Control

The effect of diet on development of the endocrine pancreas during pregnancy and early post-natal period play a key role in the development of glucose regulation and risk of glucose intolerance in later life. Morphogenesis of the endocrine pancreas tracks a similar direction and sequence in all mammals [192]. In the rat, late pregnancy and the early post-natal period are the crucial times in the development of the pancreas [193,194]. Insulin release from fetal β-cells is initially more responsive to amino acids than glucose. However, during pregnancy and through apoptosis the original β-cells will be replaced by new islet cells that are more sensitive to glucose and response during the acute first phase of insulin release [195]. In the human, pancreatic development starts at ~10 week gestation. This process continues not only during gestation but also the phase of remodeling of the islets that starts in late gestation continues to at least 4 years of age [192].

A low-protein gestational diet decreases the number of β-cells and insulin content in the fetal pancreatic islets. It also reduces the proliferation of the islet cells and increases apoptosis [196,197,198,199,200]. Although both energy restricted and low protein diets alter pancreas development and consequently glucose metabolism in the offspring, the mechanisms by which a low protein or energy restriction triggers pancreatic development are different. Protein restriction influences the proliferation of existing β-cells [199] whilst, energy restriction influences cellular neogenesis [185]. A low-protein gestational diet also decreases vascularization of the pancreas and decreases insulin secretion by the fetal pancreas in response to arginine and taurine [141,186,201].

A low protein diet during postnatal life also has a robust effect on pancreatic development. As would be expected, extending an isocaloric low-protein diet throughout gestation and post-natal period caused reduced pancreatic β-cell mass and their insulin content with smaller islets. It also resulted in downregulation of β-cell proliferation and upregulation of β-cell apoptosis in rat offspring at 3 week of age [199]. Consistently, exposure to low protein diet either through gestational post-natal period or only during post-natal period decreased the number but increased the size of islets in pancreas in offspring in both groups when compared with those offspring born to dams fed a low protein diet only during pregnancy [196]. However, low-protein diet fed throughout gesattion and postnatal life resulted in a decrease in islet blood vessel density and also in pancreatic insulin content and pancreatic and islet blood flow [202]. In addition, a reduction in insulin secretory response to both glucose and amino acids of islets was observed from 3-mo-old offspring [203]. At 3 mo of age in rats born to dams fed low protein diet throughout gestation and post-natal period, a decrease in insulin response to an oral glucose challenge was observed [86]. When low protein diets are prolonged in the post-natal period, it resulted in persistent pancreatic malfunction during post-natal period. It may suggest that low protein maternal diet has an adverse effect on endocrine pancreatic function and it could be due to an irreversible alteration during development. However, the fact that the isocaloric low protein diet naturally is higher in carbohydrates than the control as well as lower in protein may also have an independent effect on glucose metabolism.

More recently the role of muscarinic acetylcholine receptor (mAChR) subtypes alterations which may impair beta cell functioning in the adult offspring of dams fed a protein-restricted diet is studied. There are five mAChR subtypes. In investigating the effects of beta cell functioning, researchers have primarily focused on the role of the M_2_mAChR and M_3_mAChR subtypes. Upon activation the M_2_mAChR subtype blocks acetylcholine (ACh) insulinotropic action upon ACh binding, whereas M_3_mAChR subtype is the major functional receptor subtype that is expressed and responsible for the cholinergic insulinotropic effect in pancreatic islet cells. Based on these effects, Olveira *et al.* investigated the function and composition of the mAChR subtypes M_2_ and M_3_ in the pancreatic islets of adult offspring of rats that were protein malnourished while lactating. Results suggest that protein expression of M_2_mAChR in the islet of the low protein lactation offspring rats were increased by 57% compared with the normal protein lactation fed offspring; nevertheless, the protein levels of M_3_mAChR were reduced by 53% in the islets of the low protein lactation fed offspring compared again with the normal protein lactation fed offspring. Overall, these results showed a 1:3 fold higher protein expression of M_2_mAChR than of M_3_mAChR in pancreatic islets isolated from normal rats compared to the of 4:6 fold higher ratio of protein restricted rats. These results suggest that a possible cause for Ach-potentiated insulin secretion dysfunction may be caused by an overabundance or under abundance of mAChR subtypes that develops as a result of protein malnutrition in early development. Authors suggested that the low protein expression and low function of M3mAChR in pancreatic beta cells as a result of protein malnutrition early in life could lead to a low glucose insulinotropic effect, resulting in dysfunctional glucose metabolism [204].

## 6. The Role of the Timing of the Exposure to Diet

The timing of the exposure is also critical in determining the effect of maternal diet on phenotype of offspring.

*High protein diet:* Maternal high protein/low carbohydrate diet (40% protein *w*/*w*) was more instrumental in altering skeletal muscle growth and muscle fiber energy pathways when fed during lactation than fed during pregnancy in mice. The effects of a maternal high protein/low carbohydrate diet fed during lactation but not during pregnancy, negatively affected the skeletal muscle growth (myofiber size and number) of the offspring and also caused a transient adaptive shift towards oxidative metabolism (increased isocitrate dehydrogenase activity) at the expense of anaerobic glycolytic muscle metabolism (decreased lactate deyhydrogenase activity) without changing muscle fiber type composition. These results suggest that the preference for the oxidative over the anaerobic glycolytic pathway of skeletal muscle metabolism is in response to a pre-weaning high protein diet which caused alterations in skeletal muscle fiber size and number [205].

*Low protein diet:* In a similar study, goats were exposed to protein and energy restriction during late gestation. Pregnant goats were assigned to 3 diets during late pregnancy (gestation day 90 to parturition): Control, 40% protein restriction and 40% energy restriction. Offspring born to mothers fed either restricted protein or restricted energy diet had lower birth weight and also lower weights of thymus, heart, abomasums and small intestine, as proportion of body weight compared with those born to mothers fed an adequate diet. However, the difference disappeared after six weeks of nutritional recovery. Interestingly, offspring born to inadequate diets groups had a higher growth rate compared with those born to mothers fed an adequate diet. This observation can be explained based on “Catch-up growth” theory. However, whether the effect of malnutrition during late gestation on offspring’s growth is reversible through a nutritional recovery period after birth or this “catch-up growth” will have a negative impact on health of offspring in later life needs further investigation [206]. Moreover, it is not possible to understand whether these results are attributed to either calorie restriction, protein restriction or their interactive effects.

The interaction between late-gestational and post-weaning diets has also been studied in minks. Male offspring born to dams were fed either a normal- or low-protein late-gestational diet (21.2 ± 3.3 days before parturition) and received either normal- (31% of metabolizable energy (ME) from crude protein (CP)) or low-protein post-weaning diet (19% of ME from CP) until 50 weeks of age. A post-weaning low protein diet reduced the growth rate in male offspring and presented with lower liver, pancreas and kidney weight. Plasma IGF-1 concentrations at 8 and 25 weeks, the incidence of hepatic lipidosis at 25 weeks and body fat were also higher in offspring fed a low protein post-weaning diet compared with those fed a post-weaning normal protein diet. This effect was independent from their maternal dietary background [207].

Moreover, there is increasing evidence supporting the role of protein content of maternal diet during the periconceptional period in development of chronic diseases in adulthood. Dams fed a low protein diet during the periconceptional period delivered offspring with altered growth and adiposity. When mice were fed a low protein diet (9%) exclusively during oocyte development and then fed regular chow duration gestation, a significant enhanced growth phenotype in female offspring at 6 months of age was observed and it was maintained until 1 year of age, despite their smaller birth size [208]. These findings suggest that in female offspring born to dams fed low protein diets during oocyte development are programmed to up regulate energy storing and down regulate energy utilizing pathways in adipose tissue during catch up growth. Additionally, offspring from these test sets displayed significant elevated SBP at 52 weeks of age compared with the control test set [209]. In yet another similar study, adult offspring of mice fed a low protein diet (8.4% protein diet) 8 weeks prior to conception and then regular chow during gestation (starting at day 0 of conception), had altered fat deposition and gut dimensions. They were significantly shorter and lighter than those born to mothers fed a normal (21.5% *w*/*w*) or high protein diet (44.2% *w*/*w*) during preconception [209].

Overall, low protein diets fed during 8 weeks prior to conception resulted in shorter but thicker guts of offspring, which may suggest that the intestines in the offspring are affected in the same manner as the intestines of the mice actually consuming the diets themselves supporting the predictive adaptive response hypothesis for maternal preconception diets. This altered intestine (smaller and thicker dimensions) give the offspring the ability to possess a higher amount of absorptive tissue per unit length of gut, which could possibly lead to a higher absorption rate of fat, protein, carbohydrates, and calories leading to an obese phenotype [209].

More recently the time-dependent effect of perinatal maternal protein restriction on insulin sensitivity and energy substrate oxidation in adult male offspring of rats has been studied. Agnoux *et al.* suggest metabolic flexibility/inflexibility depends on when the protein malnutrition occurs: (1) when a standard diet is fed to male rats born under IUGR (intrauterine growth restriction), insulin secretion is impaired, whereas whole body insulin sensitivity is preserved; (2) in IUGR born rats that experience catch up growth, the fasting substrate switch is impaired when exposed to a standard diet, and energy storage is altered when exposed to a high energy diet; (3) in IUGR rats exposed to slow postnatal growth, mitochondrial beta oxidation is impaired when given an typical western diet; and (4) normal born rats with a slow postnatal growth rate are protected against fat accretion or insulin sensitivity loss when exposed to a western diet. These results suggest that the timing of maternal protein restriction leads to significant and specific changes in insulin sensitivity and metabolic alterations in the adult offspring [210].

The effect of proteins in maternal diet on blood pressure may also be a time-dependent effect of perinatal maternal protein insult on blood pressure. A recent investigation suggests an interactive effect of maternal diet during late gestation (second half of pregnancy) plus lactation and post-weaning diet on programming of blood pressure in rats. Offspring born to dams fed a low protein post-weaning diet (6%) were hypertensive compared with those fed a post-weaning normal protein diet (20%) and this effect was independent from their maternal dietary background (either low protein (6%) or normal protein diet (20%). Authors suggested that the effect of maternal protein deprivation on blood pressure can be normalized by a normal post-weaning diet. Authors also suggested that the mechanisms by which maternal and post-weaning diets influence blood pressure in offspring are different. More research is needed to determine exactly how these blood pressure regulatory mechanisms differ in relationship to a time dependent protein insult [211].

*Source of protein:* In another study, Jahan-mihan *et al.* showed that the effect of source of protein (casein- *vs.* soy protein-based diet) in maternal diet on food intake, body weight and also glucose metabolism is magnified when maternal diet was extended from gestation alone to gestation and lactation in male offspring of rats [23,24,25].

## 7. Sex Differences in Developmental Programming of Maternal Dietary Proteins

### 7.1. Regulation of Body Weight and Composition

*Low Protein Diet:* The results from studies investigating the effect of maternal diet on body weight and body composition provide some evidence indicating sex-dependent effect of dietary proteins. A low-protein gestational diet (90 g/kg: 9% protein diet) resulted in enhanced growth of fetus until day 20 of gestation (term = 21 day gestation) and it was followed by a growth restriction period in the last 2 days of gestation resulting in a tendency to be of low or low-normal birth weight in pups [71]. In another study, while the female offspring born to mothers fed the normal protein diet during preconception had the largest fat deposits, the male offspring born to mothers fed a low protein diet (8.4% *w*/*w*) during preconception had the largest fat deposits particularly in subcutaneous fat mass [209]. Moreover, protein content of the diet fed during preconception period influenced the development of the gastro-intestinal tract: Adult male offspring born to mice dams who were fed a low protein diet during the preconception period had a shorter total gut length compared to those born to dams fed a normal or high protein diet during the same period. Total gut mass of offspring was also higher when mothers were fed a low protein preconception diet compared with other treatment groups, again, specifically in male offspring [209].

*High Protein Diet*: When dams were fed a high protein (40% of total calorie) compared to normal protein (20% of total calorie) diet during pregnancy and lactation, no effect of maternal diet on birth weight, energy expenditure, glucose tolerance, and plasma lipid levels of the offspring was observed. However, the high protein diet resulted in higher blood pressure and glomerulosclerosis only in male offspring, whereas increased food efficiency, higher body weight, and increased fat pads characterized in the female offspring [20]. Female offspring also exhibited a higher body weight at the beginning of puberty, persisting until the end of the experiment (week 22) [20]. In another study, offspring born to rat dams fed a high protein diet (40% of total calorie) during pregnancy presented with lower body weight on day 2 of life compared with controls (20% of total calorie) and had greater fat mass and decreased energy expenditure at week 9 of age. Postnatal high protein diet alone had no effect on body composition or metabolic rate [22].

### 7.2. Regulation of Blood Glucose

*Low Protein Diet:* There is substantial evidence indicating that the dams’ diet affects glucose metabolism in the offspring in a sex-dependent manner [86,165,212,213,214,215] including offspring of dams fed high and low protein diets [165,201]. For example, impaired glucose tolerance was found in adult females and their insulin response to an oral glucose preload was low if they were born to rat dams fed a low protein (8% protein w/w) diet during gestation [201]. In contrast, male but not female Wistar rats born to dams fed low protein (8% *w*/*w*) diets were more hyperinsulinemic and insulin resistant at 20 weeks of age [165].

*Source of Protein:* Protein source in a nutritionally adequate diet (20% *w*/*w*) during gestation and lactation influenced glucose metabolism in offspring of rats in a sex-dependent manner. Dams’ soy protein-based diet resulted in higher fasting glucose, glucose response to glucose preloads and the HOMA-IR index, which are indicators of insulin resistance, in male offspring compared with dams’ casein-based diet [23,24], while had no effect in females at week 14 after weaning [25]. Because female rats develop insulin resistance later in life (e.g., 21 months) than males [214] it may be suggested that 14 week duration post-weaning was too short to show an effect in females.

### 7.3. Regulation of Blood Pressure

Sex hormones are concerned as potential mediators of sex-specific fetal programming of cardiovascular risk [49,215,216]. In intrauterine growth restriction (IUGR) rats, hypertension in male is associated with a twofold increase in circulating testosterone [217] implicating the importance of testosterone in the etiology of IUGR-induced hypertension. Interestingly, estradiol replacement in ovariectomized female IUGR offspring reverses the increase in blood pressure that occurs after a loss of ovarian hormones in the female IUGR rat [218]. Moreover, estrogen exerts a protective effect in response to chronic Angiotensin II by altering the vasoconstrictor/vasodilator balance [218].

In human, the sex-dependent effect of low birth weight on blood pressure has been investigated by several studies: In one study on 600 children (3–6 year of age) in China, the inverse relationship between birth weight and systolic blood pressure (SBP) was stronger in boys compared with girls [219]. However, in another study on 4000 youth (15 year of age) in Sweden noted a stronger relationship between birth weight on SBP in girls than in boys [220]. It may suggest that variation in age can determine the interactive effect of sex and birth weight on SBP.

*Low Protein Diet:* In rats, low maternal protein restriction (9% *vs.* 20%) induced hypertension in a sex dependent manner (only in male offspring) [221,222,223]. However, this effect was not through a testosterone-dependent mechanism when it was a 50% reduction in maternal protein intake [224]. Interestingly, a more severe protein restriction (5% *vs.* 20%) induced hypertension in both females and males [224].

*Source of Protein:* Additionally as seen in blood glucose regulation and body composition, source of protein in a nutritionally adequate dams’ diet influences systolic and diastolic blood pressure and pulse rate in male offspring and systolic blood pressure in female offspring. Diastolic blood pressure and pulse rate in males were higher in offspring born to dams fed the soy protein-based diet compared with those born to dams fed the casein-based diet; and a higher systolic blood pressure was also found in soy protein based fed females [24].

### 7.4. Regulation of Liver Triglyceride and Cholesterol Metabolism

*Low Protein Diet:* There is considerable evidence suggesting that maternal protein restriction during pregnancy and lactation in rats may induce long-term reduction in hepatic lipid content, especially in a sex-dependent manner. Maternal low protein diet (8% *w*/*w*) fed during pregnancy and lactation cause a noteworthy reduction in liver triglyceride content in male offspring at day 65 and at day 150. However, these results were not seen in the female offspring. Additionally, maternal low protein diet did not affect liver cholesterol content in 65 day old male offspring but was significantly reduced at 150 days. Maternal low protein diet did not affect liver cholesterol content in females at any age. This suggests that the effect of maternal protein restriction on the hepatic content of lipids in the offspring depends on the type of lipid and also depends on the sex. Authors suggest that sex hormones may play a protective role in female offspring by preventing diet alteration induced hepatic lipid metabolic changes [225]. In another study, mouse female offspring born to dams fed a low protein diet (9% casein *w*/*w*) showed a significant enrichment of genes involved in lipid metabolism, implying that *in utero* protein restriction in female offspring leads to physiologic effects observed in glucose and fatty acid metabolism. However, a prenatal maternal low protein diet was shown to have unfavorable effects in maintaining insulin sensitivity in female offspring fed a postnatal high fat diet (40% cal) compared with control females fed a low fat diet [226]. Female offspring fed a high fat postnatal diet also showed lower insulin sensitivity, unchanged Metabolic Clearance Rate (MCR), and higher Rate of Appearance (RA) of glucose compared with offspring fed a low fat diet. However, male offspring born to dams fed low protein maternal diet during pregnancy and fed a postnatal high fat diet, showed lower insulin sensitivity, reduced MCR, and unchanged RA of glucose compared with low-fat diet–fed male offspring. This finding indicates the male offspring develop peripheral insulin resistance later in life whereas females are more prone to develop hepatic insulin resistance [226]. Further studies needed to unveil the underlying mechanisms.

## 8. Summary

In summary, both observational and experimental studies showed that dietary proteins in maternal diet are major modifiers of the development of regulatory systems in the offspring *in utero* and post-natally and their effect is time- and also sex-dependent. However, it is critically important to consider the fact that any change in protein content of maternal diet (high or low protein diet) will change the whole dietary composition and will change other macronutrients’ contents of experimental diets proportionally. For example, the effect of high protein, low carbohydrate diets that are being used increasingly in developed countries on developmental programming can be due to both higher protein and also lower carbohydrate contents of these diets that needs further study. Although changes in protein content and also source in maternal diets influenced the fetal development and health outcomes in the offspring, extremes of protein intake are less likely concern of women in developed countries. More likely, protein source may be a significant factor because many differences in health outcomes are observed in vegetarian compared with omnivores [227,228]. Some of these differences may be attributed to source and composition of proteins consumed during pregnancy and infancy. The fact that adding individual amino acids to maternal low protein diets diminishes their detrimental effects suggests a role for the source of protein. Finally, physiological effect of protein arise from their amino acid composition, BAPs and digestion kinetics are potential factors affecting development beyond their nutritional role of providing sufficient amino acids for growth.

## 9. Future Direction

The role of both high and low protein maternal diets in the development of phenotype of offspring has been studied extensively. However, the role of other macronutrients’ content of high and low protein maternal diets needs more study. For example, comparison can be made between a low protein, low fat, high carbohydrate and a low protein, high fat, low carbohydrate diet. Moreover, to examine the mechanisms by which the source of protein in maternal diet alters health of offspring, the role of characteristics of proteins including amino acid composition, digestion kinetics, amino acid sequence and finally potential bioactive peptides can be studied. In addition, an urgent care must be given to the effect of maternal obesity and its effect on offspring’s health. It is eminent that maternal obesity during pregnancy increases the risk of gestational diabetes in mothers and characteristics of metabolic syndrome in offspring. To our knowledge, no study has examined the effect of quantity and source of protein consumed during pregnancy in obese mothers on both mothers’ health and their children. It is particularly important because more than two-thirds of women ages 20–39 in the United States are overweight and or obese and half of them are obese [229].
